# Bilateral renal angiomyolipoma with venous invasion: A case report

**DOI:** 10.1016/j.radcr.2024.04.006

**Published:** 2024-05-15

**Authors:** Fatima Emam, Rani Hammoud, Akram Twair, Mohamed lamier Mukhtar Hussein

**Affiliations:** a Radiology Department, Hamad general hospital, Hamad medical corporation (HMC), Doha Qatar; b Otolaryngology Department, Hamad general hospital, Hamad medical corporation (HMC), Doha Qatar

**Keywords:** Renal angiomyolipoma, AML, Inferior vena cava, Renal vein, Thrombus

## Abstract

Renal angiomyolipoma (AML) is a rare benign tumor of the kidney, often detected incidentally on radiological images as the presence of macroscopic fat characterizes them. In the majority of the cases, they are usually sporadic. Despite their benign nature, venous invasion, a rare occurrence in renal AMLs, poses management challenges. We present a case of bilateral renal AML in a 52-year-old female with a right renal vein and hepatic inferior vena cava invasion.

## Introduction

Renal angiomyolipoma (AML) is a rare benign renal tumor constituting a mere 0.3%-3% of all renal neoplasms and is typically encountered incidentally through radiological imaging [1], [2], [3]. These tumors unveil a complex architecture characterized by the presence of highly vascular tissue that contains both smooth muscle and adipose elements [2]. While renal AMLs are sporadic in 80% of the cases, they also exhibit a notable association with specific conditions such as tuberous sclerosis and pulmonary lymphangioleiomyomatosis, encompassing approximately 20% of instances [3,4]. Clinical presentation of renal AML may vary; some patients are asymptomatic, while others may experience symptoms such as flank pain, gross hematuria, and, in severe cases, retroperitoneal hemorrhage [3]. Radiological renal AML is characterized by the presence of macroscopic fat, which can be seen in all radiological modalities [5,6]. However, despite their benign nature, renal AMLs can occasionally demonstrate unexpected behavior, such as venous invasion, and such occurrence carries clinical implications on patient management [7]. In this paper, we are reporting a case of bilateral renal AML in a 52-year-old female with renal vein and inferior vena cava invasion.

## Case presentation

A 52-year-old female patient with no past medical or surgical history presented to the emergency department with intermittent bilateral flank pain and dysuria. She had no fever or other urinary symptoms. She had no signs or symptoms of tuberous sclerosis complex (TSC). Her physical examination, general hematology, and blood chemistry were unremarkable. Urine analysis indicated a trace of leukocytes; otherwise, it was unremarkable. A bedside ultrasound revealed a right renal mass; thus, a contrast-enhanced abdominal computed tomography (CT) scan was arranged.

**Fig. 1:**
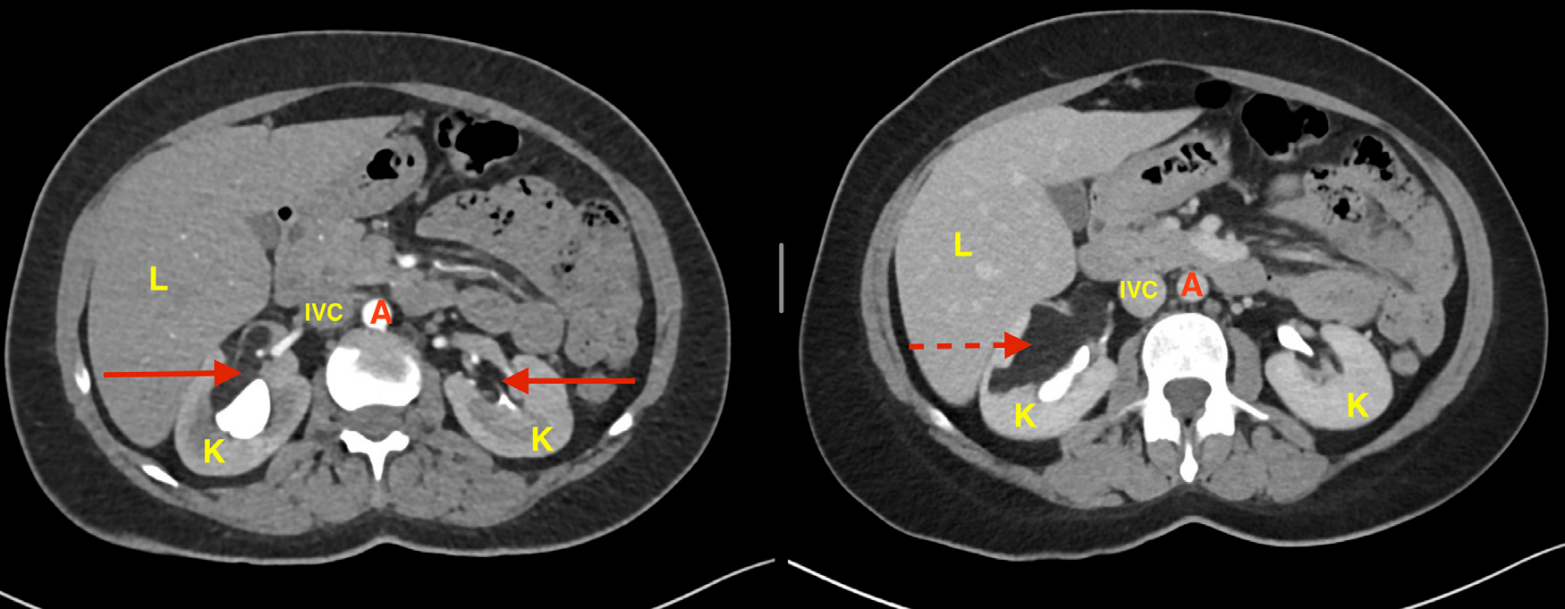
CT abdomen axial cut with contrast at 2 levels. • K: kidney. • L: liver. • A: abdominal aorta. • IVC: inferior vena cava. • Red solid line arrows demonstrating the right and left fat rich renal masses. • Red dotted line arrow showing the right renal mass involving the lateral cortex.

The CT abdomen, with contrast, revealed bilateral fat-density renal masses, the largest seen in the right renal sinus, with focal involvement of the lateral cortex. The lesion measures 6.7 × 3.6 × 4.6 cm with focal dilatation of the calyceal system, mainly the upper calyx, likely due to compression effect (Fig. 1).

**Fig. 2:**
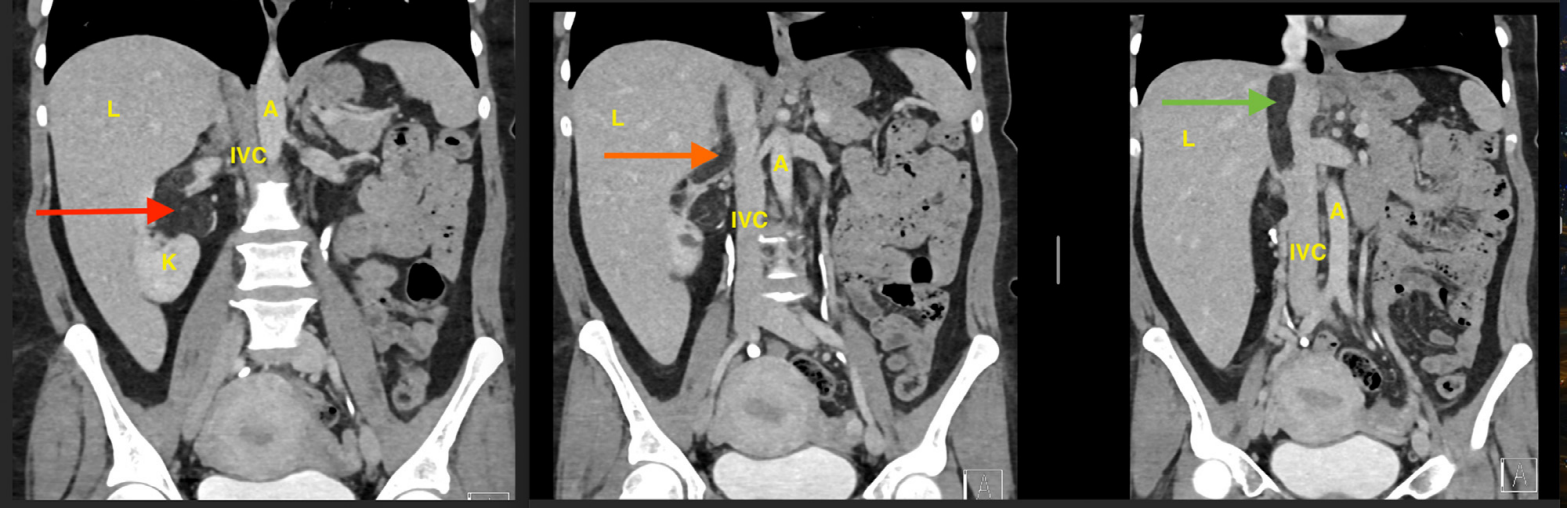
CT abdomen axial cut with contrast at 3 levels. • K: kidney. • L: liver. • A: abdominal aorta. • IVC: inferior vena cava. • Red solid-line arrows: the right renal mass abutting the pelvi-ureteric junction • Orange solid-line arrow: thrombus in the right renal vein • Green solid-line arrow: thrombus in the inferior vena cava.

On Abdominal Angio-scan, the tumor is seen extending medially abutting the pelvi-ureteric junction, pushing it medially and invading the anterior branch of the right renal vein, extending to the main renal vein and hepatic inferior vena cava (IVC), subsequently (length of tumor thrombus inside the IVC about 7 cm), no supradiaphragmatic extension (Fig. 2)

The other lesion is seen in the lateral cortex of the left renal midpole, measuring 4 mm with no vascular involvement on abdominal angio-scan.

Additionally, a CT Chest high resolution was done, which was unremarkable, with no evidence to suggest pulmonary lymphangioleiomyomatosis or any other pathologies.

A multi-disciplinary team meeting was conducted where the decision of right radical nephrectomy and thrombectomy was taken with the patient, considering the risk of pulmonary embolism due to renal vein and IVC thrombus. Unfortunately, the patient differed from surgery and lost follow-up.

## Discussion

Renal AML is a rare bengin tumor that was first described by Grawitz in 1900 [2]. It is highly vascular with a large amount of adipose tissue, which occurs more in females, possibly due to estrogenic effects [2,4]. The diagnosis of renal AML heavily depends on imaging modalities, and a biopsy is not generally required [8]. AML usually appears as fat-rich tumors; however, they can be fat-poor or fat-invisible in about 5% of cases [5,6]. The primary diagnostic feature of classic AML (fat-rich) is the identification of a significant amount of adipose tissue seen in CT scans [9]. In cases of fat-poor or fat-invisible AML, there is insufficient adipose tissue, and an magnetic resonance Imaging is usually needed for the diagnosis [4,9]. In selected cases where there is high suspicion of active bleeding, a contrast-enhanced CT scan may be indicated [4,9]. Although renal AML is a benign tumor, it may show signs of invasion as it can extend into the surrounding perirenal fat, renal sinus, or nearby organs [10]. Some reports show tumor thrombi extending through the renal vein and into the inferior vena cava [11,12]. Our patient had bilateral renal masses with the right side measuring more than 6 cm associated with right renal vein invasion extending to the IVC in the subdiaphragmatic region. Renal AML that are either bilateral or Large (>3 cm) tend to be seen in patients with tuberous sclerosis or pulmonary lymphangioleiomyomatosis [13,14]. Although our patient meets these qualities, she had no medical history of tuberous sclerosis or any neurological findings, in addition her chest CT scan showed no lung pathology.

Given the high risk of thromboembolism in patients with renal AML associated with venous invasion, radical nephrectomy and thrombectomy are usually the chosen management [7]. Alternative treatment approaches, such as partial nephrectomy or embolization, may be considered in select cases [2]. Long-term follow-up data on patient outcomes would provide valuable insights into prognosis and potential complications, though currently unavailable in this report.

## Conclusion

This case report underlines the rarity of venous invasion in renal AML. By highlighting this unique presentation, our report contributes to the existing literature on renal AML with venous invasion, informing clinical practice and guiding future research endeavors.

## Patient consent

Written informed consent for publication was obtained to publish this case report.
